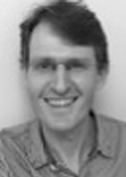# Mechanistic Investigations into the Application of Sulfoxides in Carbohydrate Synthesis

**DOI:** 10.1002/chem.201503504

**Published:** 2016-01-07

**Authors:** Martin A. Fascione, Robin Brabham, W. Bruce Turnbull

**Affiliations:** ^1^ York Structural Biology LabDepartment of ChemistryUniversity of YorkHeslington RoadYorkYO10 5DDUK; ^2^ School of Chemistry and Astbury Centre for Structural Molecular BiologyUniversity of LeedsWoodhouse LaneLeedsLS2 9JTUK

**Keywords:** carbohydrates, glycosylation, mechanism, organic synthesis, sulfoxides

## Abstract

The utility of sulfoxides in a diverse range of transformations in the field of carbohydrate chemistry has seen rapid growth since the first introduction of a sulfoxide as a glycosyl donor in 1989. Sulfoxides have since developed into more than just anomeric leaving groups, and today have multiple roles in glycosylation reactions. These include as activators for thioglycosides, hemiacetals, and glycals, and as precursors to glycosyl triflates, which are essential for stereoselective β‐mannoside synthesis, and bicyclic sulfonium ions that facilitate the stereoselective synthesis of α‐glycosides. In this review we highlight the mechanistic investigations undertaken in this area, often outlining strategies employed to differentiate between multiple proposed reaction pathways, and how the conclusions of these investigations have and continue to inform upon the development of more efficient transformations in sulfoxide‐based carbohydrate synthesis.

## Introduction

The widespread use of sulfoxides in organic chemistry is a result of their rich and varied reactivity^1^ showcased by an enviable plethora of reactions. Well‐studied examples include the use of dimethyl sulfoxide in the oxidation of alcohols,^2^ the activation of sulfoxides in Pummerer‐type reactions,^3^ and pericyclic reactions of sulfoxides, such as the Mislow–Evans rearrangement.^4^ However, few fields have benefited more from the diverse chemical capabilities of sulfoxides than modern synthetic carbohydrate chemistry,^5^ for which they often play integral roles as leaving groups, or as activating agents in high yielding glycosylation reactions. An all‐encompassing review of the use of sulfoxides in carbohydrate chemistry has been forsaken here in favour of an in‐depth analysis of the elegant mechanistic investigations performed in this area, which have begun to underpin many of the contemporary theories regarding stereoselectivity and efficiency in challenging sulfoxide‐based carbohydrate synthesis. Included will be a discussion on the use of glycosyl sulfoxides as glycosyl donors, as well as the application of sulfoxide reagents in dehydrative glycosylations, glycal activation and thioglycoside donor activation.

## Glycosyl sulfoxides

**Scheme 1:**
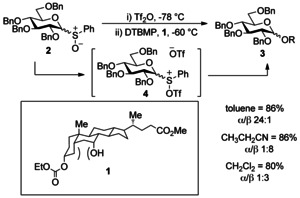
The challenging glycosylation of a deoxycholic ester is feasible using sulfoxide‐based glycosyl donors. DTBMP=2,6‐di‐*tert*‐butyl‐4‐methylpyridine.

The use of thioglycoside donors has been widespread since their introduction by Ferrier.^6^ The next substantial step forward in the use of thioglycoside derivatives came from Kahne and co‐workers^7^ who originally developed the concept of using a sulfoxide glycosyl donor after unsuccessful attempts to glycosylate deoxycholic ester derivative **1** (Scheme 1), in which the target axial alcohol is very unreactive due to 1,3‐diaxial steric hindrance. Sulfoxide glycosylation reactions with benzylated donor **2** and deoxycholic ester **1** afforded glycoside **3** in excellent yield, in a number of different solvents (Scheme 1).

**Scheme 2:**
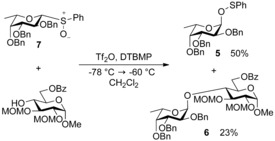
At sufficiently low temperatures, glycosyl sulfenate **5** can be isolated from glycosylations involving glycosyl sulfoxides. MOM=methoxymethyl ether.

Activation of the sulfoxide was achieved with triflic anhydride at −78 °C, and proceeded through putative sulfonium triflate species **4**. Further examples with benzyl and pivaloyl‐ protected donors were also high‐yielding, and included the first example of glycosylation of an amide nitrogen atom, using trimethylsilyl acetamide—an early demonstration of the potential utility of glycosyl sulfoxides as novel glycosyl donors. Kahne and co‐workers noted the glycosylation of less reactive trimethylsilyl acetamide stalled at −78 °C, but re‐initiated between 0 °C and ambient temperature over 12 h.^7^ Having previously demonstrated the reactivity of glycosyl sulfoxides at low temperatures, the authors postulated any reactive intermediates present at −78 °C would decompose at higher temperatures. This implied that glycosylation at the higher temperatures occurred via an unidentified more stable intermediate. After further investigation, this unknown intermediate was subsequently assigned as a glycosyl sulfenate as the sulfenate **5** and disaccharide **6** were isolated in a 2:1 ratio (Scheme 2) following activation of fucose donor **7** at −60 °C.^8^ Application of glycosyl sulfenates as donors had previously been performed at 0 °C;^9^ therefore, the isolated glycosyl sulfenate **5** seemed a likely candidate as a reactive intermediate in the sulfoxide reactions at higher temperatures.

**Scheme 3:**
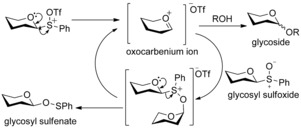
Proposed mechanism for triflic anhydride activated glycosylation of sulfoxide donors, accounting for the glycosyl sulfenate byproduct.

Subsequently, formation of glycosyl sulfenates from glycosyl sulfoxides was achieved using catalytic triflic anhydride.^8^ Based upon this observation a mechanism to account for formation of both glycosides and glycosyl sulfenates in sulfoxide glycosylations was proposed (Scheme 3). Following these mechanistic insights, Kahne and co‐workers developed a strategy to scavenge byproducts in the sulfoxide glycosylation reaction using 4‐allyl‐1,2‐dimethoxybenzene,^10^ an improvement that aided their program of challenging synthetic endeavours including the synthesis of the blood‐group antigens,^11^ the calicheamicin oligosaccharide^12^ and the ciclamycin trisaccharide.^12^

## Stereoselective synthesis of β‐mannopyranosides and α‐glucopyranosides

**Scheme 4:**
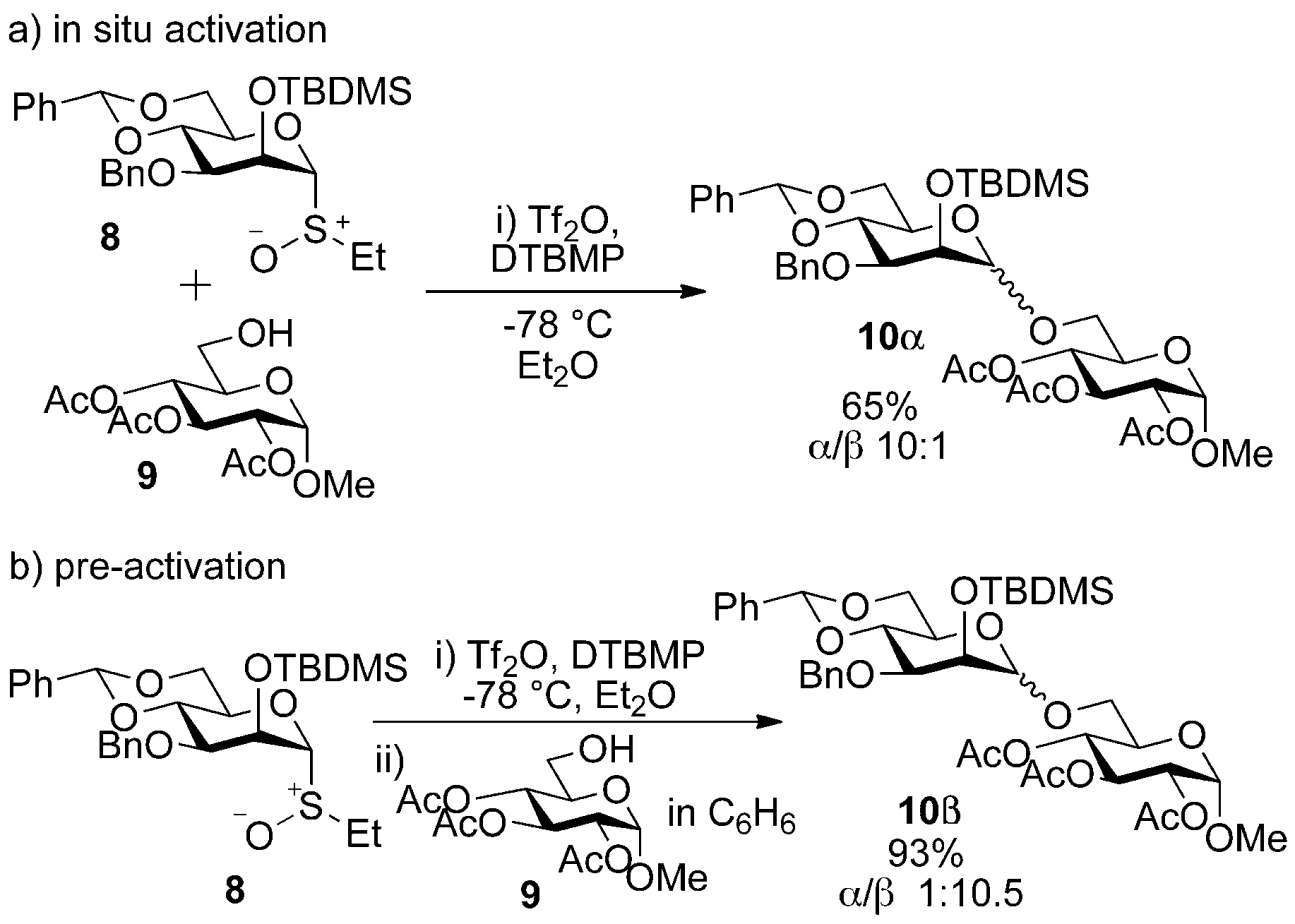
Dependence of stereoselectivity upon order of addition of glycosyl acceptor versus activating agents. TBDMS=*tert*‐butyldimethylsilyl.

While pursuing a radical‐based solution^13^ to the ubiquitous problem of stereoselective β‐mannopyranoside synthesis,^14^ Crich and co‐workers serendipitously uncovered an unappreciated level of complexity in Kahne's sulfoxide glycosylation method.^15^ When using benzylidene acetal protected donor **8**, Crich observed that the stereoselectivity of the reaction was dependent on the order of addition of the acceptor and activating agent (Scheme 4). If donor **8** and acceptor **9** were premixed in diethyl ether and then activated with triflic anhydride, α‐mannopyranoside **10 α** was formed stereoselectively (in situ activation protocol, Scheme 4 a). However, when the donor **8** was activated with triflic anhydride in diethyl ether prior to the addition of the acceptor **9**, a complete reversal in selectivity was observed and β‐mannopyranoside **10 β** was formed stereoselectively (pre‐activation protocol, Scheme 4 b).

**Scheme 5:**
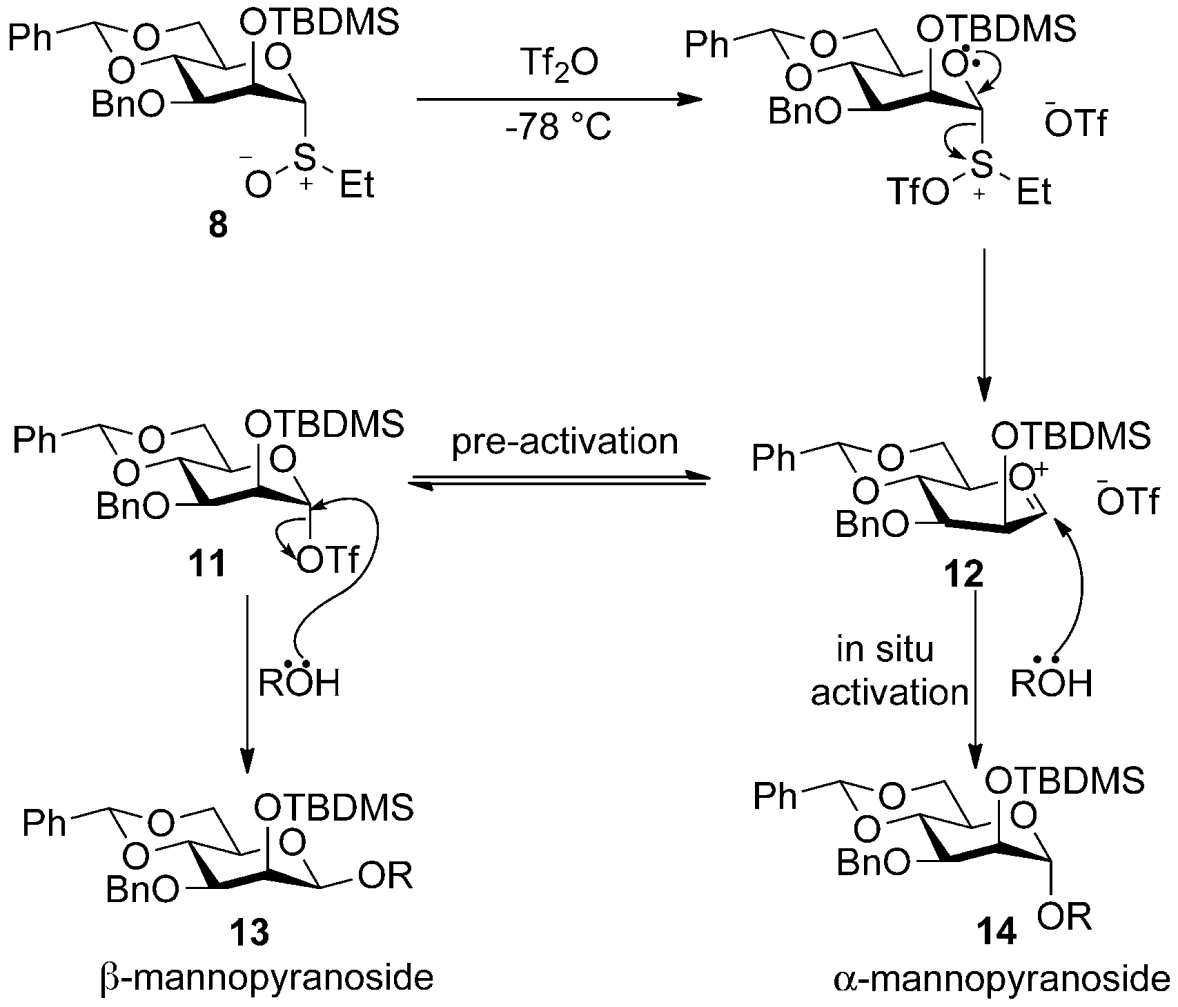
Proposed mechanisms for the formation of β‐mannopyranoside **13** and α‐mannopyranoside **14**.

The utility of this new methodology for direct β‐mannopyranoside formation was demonstrated with a number of acceptor alcohols. However, it was noted that the benzylidene acetal was essential for selectivity. When the fully benzylated equivalent donor was used the selectivity of the reaction was reduced significantly (α/β 2:1). The mechanistic rationale deployed to explain these observations involved inferring the presence of a glycosyl triflate intermediate **11** (Scheme 5).^16^ In the proposed mechanism, the fate of the oxacarbenium ion **12** depends on the order of addition of the reagents. In the absence of the acceptor (pre‐activation), a putative α‐glycosyl triflate **11** is formed which reacts with an acceptor alcohol with inversion of configuration to afford β‐mannopyranoside **13**. Alternatively, when activation occurs in the presence of the acceptor alcohol (in situ activation) the oxacarbenium ion **12** affords α‐mannopyranoside **14**.

In this hypothesis, the observed β‐selectivity arises from S_N_2‐type attack of the alcohol on the α‐triflate species **11** (glycosyl tosylates with similar reactivity had previously been disclosed).^17^

This observation was initially substantiated by increased β‐selectivities (α/β 1:13→1:32) when less bulky *O*‐2‐benzyl donor **15** was used in a less‐ionizing dichloromethane solvent. It should also be noted that other groups have established that pre‐activation of Crich's benzylidene acetal donors is not necessarily a prerequisite for β‐mannoside selectivity when glycosylations are performed in dichloromethane as opposed to diethyl ether.^18^

**Scheme 6:**
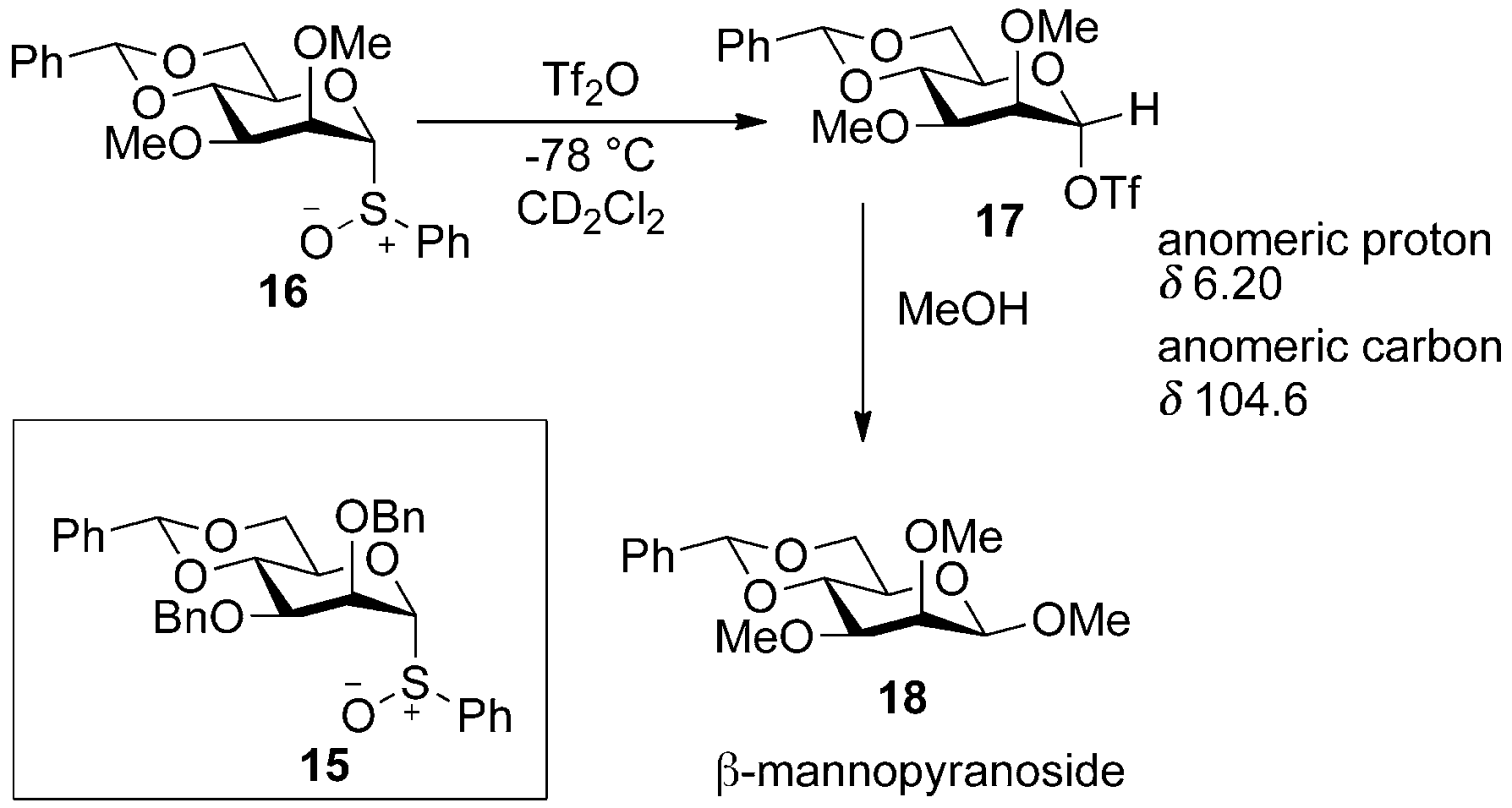
NMR studies of intermediate glycosyl triflate **17**.

Subsequent evidence for the existence of α‐triflate species came from low‐temperature NMR studies of the glycosylation reaction.^19^ Using simplified donor **16** the mechanism was probed by activation at −78 °C with triflic anhydride (Scheme 6). Within acquisition of the ^1^H NMR spectrum a new intermediate had formed with a characteristic H1 shift of *δ*=6.20 ppm, and a ^13^C NMR C1 shift of *δ*=104.6 ppm.^17^ The intermediate was assigned as glycosyl triflate **17**, and subsequently afforded β‐mannopyranoside **18** on addition of methanol.

A key point established by Crich is the necessity of the benzylidene acetal‐protecting group for β‐selective mannosylations.^16^, ^19^ This is attributed to the increased conformational constraint imposed on the sugar ring by the benzylidene acetal, which disfavours the formation of the half‐chair oxacarbenium ion,^20^ thus promoting the formation of a *trans*‐decalin‐like glycosyl triflate intermediate.

**Scheme 7:**
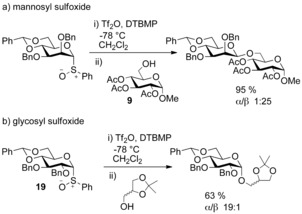
Differing selectivities in the glycosylation of mannosyl sulfoxides and glucosyl sulfoxide **19**.

An unexpected reversal of stereoselectivity was observed when glycosylation of glucosyl sulfoxide donors was performed. The authors isolated only α‐glycosides selectively (Scheme 7 b), compared to mannosyl sulfoxide donors, which afforded β‐glycosides selectively (Scheme 7 a).^21^ The benzylidene acetal protecting group was again a prerequisite for stereoselectivity (although glycosylations with glucosyl sulfoxide **19** and triflic anhydride afford α‐glucosides, better yields and selectivities were achieved by activation of thioglucosides with PhSOTf).^22^

**Scheme 8:**
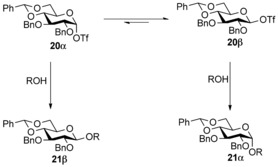
Stereoselective formation of α‐glucopyranoside **21α** by virtue of a Curtin–Hammett kinetic scenario.

The authors postulated selectivity arises from reaction of the acceptor with transient glycosyl triflates **20** (Scheme 8). The mechanistic rationale used for the gluco series differs from that of the manno series, in that the reactive intermediate is β‐glucosyl triflate **20 β** rather than α‐glucosyl triflate **20 α**. A Curtin–Hammett kinetic scheme^23^ was invoked to explain selectivity, in which the reaction proceeds through the less stable, and thus more reactive β‐glucosyl triflate **20 β**.

**Scheme 9:**
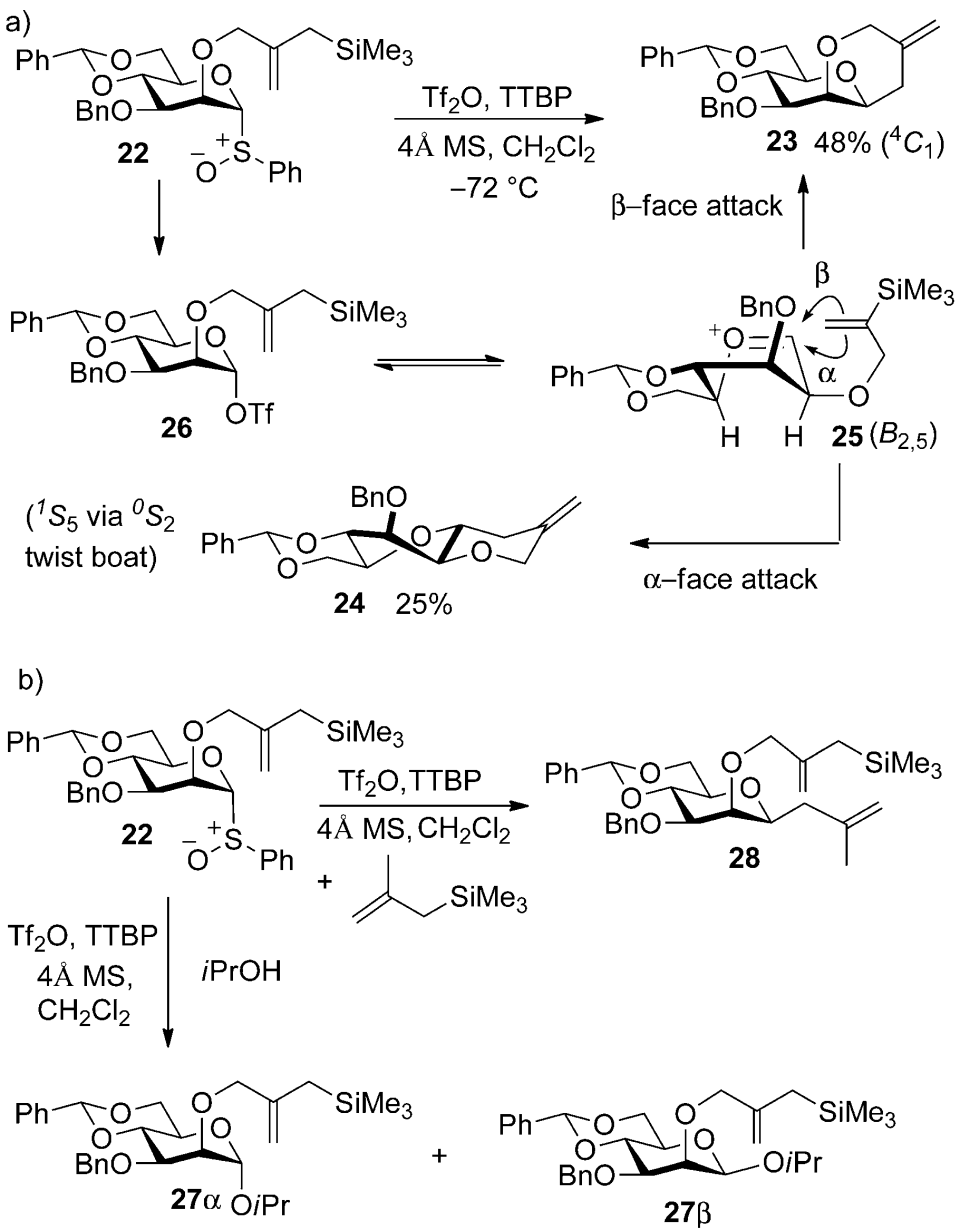
Crich's cation‐clock. a) Intramolecular Sakurai reaction of mannosyl sulfoxide **23** and b) competing *O*‐glycosylation with isopropanol, or *C*‐glycosylation CH_2_=C(CH_3_)CH_2_TMS. TTBP=2,4,6‐tri‐*tert*‐butylpyrimidine.

These initial explorations were followed up with a number of mechanistic studies on the chemistry of glycosyl sulfoxides and glycosyl triflates.^24^ However, until recently there remained a degree of ambivalence over whether the stereoselective attack on glycosyl triflates truly proceeded through an S_N_2‐like or S_N_1‐like mechanism. To jettison any ambiguity, Crich re‐tooled two classical approaches for elucidating chemical reaction kinetics—employing a cation‐clock experiment,^25^ and a natural abundance kinetic isotope study^26^ to unequivocally prove the reaction proceeds through an S_N_2‐like mechanism. Crich's cation‐clock was developed to distinguish between different mechanisms by measuring the relative kinetics between α‐ and β‐*O* and β‐*C*‐mannopyranosylations and a competing intramolecular cyclisation (Scheme 9). Following triflic anhydride activation of the mannopyranosyl sulfoxide **22**, which bears a prospective internal Sakurai nucleophile, a major **23** (β‐face attack affords the ^4^ *C*_1_ chair conformer) and minor product **24** (α‐face attack affords a ^1^ *S*_5_ twist‐boat conformer) were formed. The formation of both products was rationalised by intramolecular attack from either the α‐ or β‐face of the *B*_2,5_ twist‐boat mannosyl oxacarbenium ion **25**,^27^ which exists in equilibrium with a glycosyl triflate **26**. The authors then repeated triflic anhydride activation experiments, but rapidly followed with the addition of increasing quantities of isopropanol as a glycosyl acceptor. This reaction manifold allowed the quantification of individual mannopyranosyl anomers **27 β** and **27 α** formation with respect to the intramolecular cyclisation products **23** and **24**, as a function of isopropanol acceptor concentration. This methodology was also repeated with trimethyl methallylsilane as an external competing *C*‐nucleophile, to report on the kinetics of *C*‐glycoside formation.

The cation‐clock experiment demonstrated firstly that the ratio of formation of β‐isopropyl mannoside **27 β** to cyclised products increases as isopropanol concentration increases; therefore, the formation of β‐*O*‐mannosides is first order with respect to nucleophile concentration. Conversely, the ratios of formation of α‐isopropyl mannoside **27 α** and β‐*C*‐mannoside **28** to cyclised products did not change with increasing nucleophile concentration, and was thus deemed zeroth order overall with respect to nucleophile concentration.

**Scheme 10:**
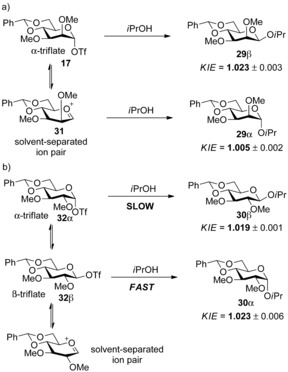
Natural abundance ^13^C NMR KIE study, on formation of a) mannopyranosides **29 α** and **29 β** and b) glucopyranosides **30 α** and **30 β**.

These results are consistent with S_N_2‐like isopropanol attack on an α‐mannosyl triflate, or an α‐contact ion pair, in accordance with Crich's earlier postulate; the formations of the α‐isopropyl mannoside **27 α**, and β‐*C*‐mannoside **28** were consistent with an S_N_1‐like isopropanol attack on an oxacarbenium ion or a solvent‐separated ion pair.25a This study was closely followed by a complementary measurement of primary kinetic isotope effects (KIEs) using natural abundance of ^13^C and very high‐field NMR spectroscopy (200 MHz for ^13^C NMR) to measure the formation of α‐ and β‐mannopyranosides and α‐ and β‐glucopyranosides via transient glycosyl triflates.^26^ A biased system facilitated erosion of the natural selectivity of the glycosylation reaction, allowing ^13^C‐1 signals of both anomeric products to be measured, using the benzylidene acetal carbon as an internal standard (Scheme 10). The ratios calculated were then compared to the same ratio in the glycosyl sulfoxide starting material. The calculated KIEs for the formation of the β‐mannopyranosides **29 β**, α‐ and β‐glucosides **30 β** and **30 α** were all in the lower range expected for a bimolecular reaction (1.03–1.08), while the KIE measured for the formation of α‐mannopyranoside **29 α** (1.005±0.002) was in the range for a unimolecular reaction (1.00–1.01). These results again provided further confirmation for the formation of β‐mannopyranosides through an exploded S_N_2‐like transition state, and α‐mannopyranosides through S_N_1‐like attack on an oxacarbenium ion or a solvent‐separated ion pair such as **31**. While formation of α‐ and β‐glucopyranosides in the analogous glycosylation reaction are also a result of bimolecular S_N_2‐like attack on glycosyl triflates, for example, **32 α** and **32 β**, once again the preference for the α‐product can be explained by inference of a Curtin–Hammett kinetic scenario, where the less stable minor β‐triflate reacts more quickly to afford the α‐anomer preferentially.

**Scheme 11:**
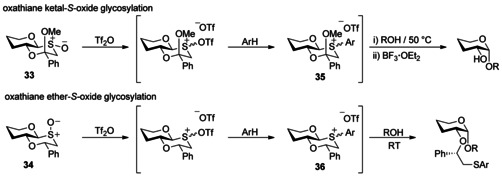
Activation of oxathiane ketal‐(*S*)‐oxide **33** and oxathiane ether‐(*S*)‐oxide **34** by umpolung *S*‐arylation. Reproduced from ref. 28b.

**Scheme 12:**
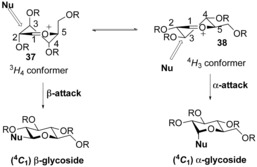
The equilibrium between the ^3^ *H*_4_ and ^4^ *H*_3_ oxacarbenium conformers **37** and **38** can govern the overall stereoselectivity of glycosylation

Our own mechanistic studies in this field of stereoselective glycosylation of glycosyl sulfoxides have been focussed upon the activation and reactivity of oxathiane‐*S*‐oxide donors **33** and **34** (Scheme 11).^28^ The *trans*‐decalin motif present in these oxathianes conferred unanticipated stability on aryl sulfonium ions **35** and **36**, to the extent that their formation could be monitored with NMR at ambient temperature, following triflic anhydride activation in the presence of electron‐rich arenes.28b All protected derivatives of the oxathiane ketal‐*S*‐oxide displayed complete α‐anomeric stereoselectivity, even at 50 °C, suggestive of an S_N_2‐like attack on the aryl sulfonium ion from the α‐face. While still highly α‐stereoselective, the oxathiane‐ether‐*S*‐oxide also afforded β‐glycosides, indicative of at least partial S_N_1‐like attack on an oxacarbenium ion, and raised the question of whether the exchange of an axial methoxy group for a hydrogen atom could effect a change in the mechanism from stereospecific S_N_2‐like attack to a highly stereoselective S_N_1‐like attack. However, DFT calculations using model structures indicated that both the oxathiane ketal and ether were equally likely to react by an S_N_2‐like mechanism, discounting this tantalising proposition. Instead calculations of the relative stability of the relevant oxacarbenium ion conformers: ^4^ *H*_3_ **38** (S_N_1‐like attack upon which affords α‐glycosides) and ^3^ *H*_4_ **37** (attack upon which affords β‐glycosides) indicate it is more likely the erosion in α‐stereoselectivity results from an increase in the population of ^3^ *H*_4_ conformers upon removal of the axial methoxy group (Scheme 12).

## Dehydrative glycosylation

**Scheme 13:**
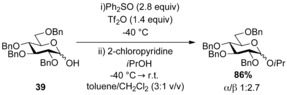
Dehydrative glycosylation using Ph_2_SO and triflic anhydride.

Sulfoxides have also been used as activating agents in glycosylation reactions to facilitate in situ formation of reactive glycosylating species. Gin and co‐workers identified sulfoxides as the ideal reagents for dehydrative glycosylation of hemiacetal donors.^29^ In a representative example, a combination of Ph_2_SO and triflic anhydride was used to pre‐activate hemiacetal donor **39** prior to the addition of a glycosyl acceptor (Scheme 13).

**Scheme 14:**
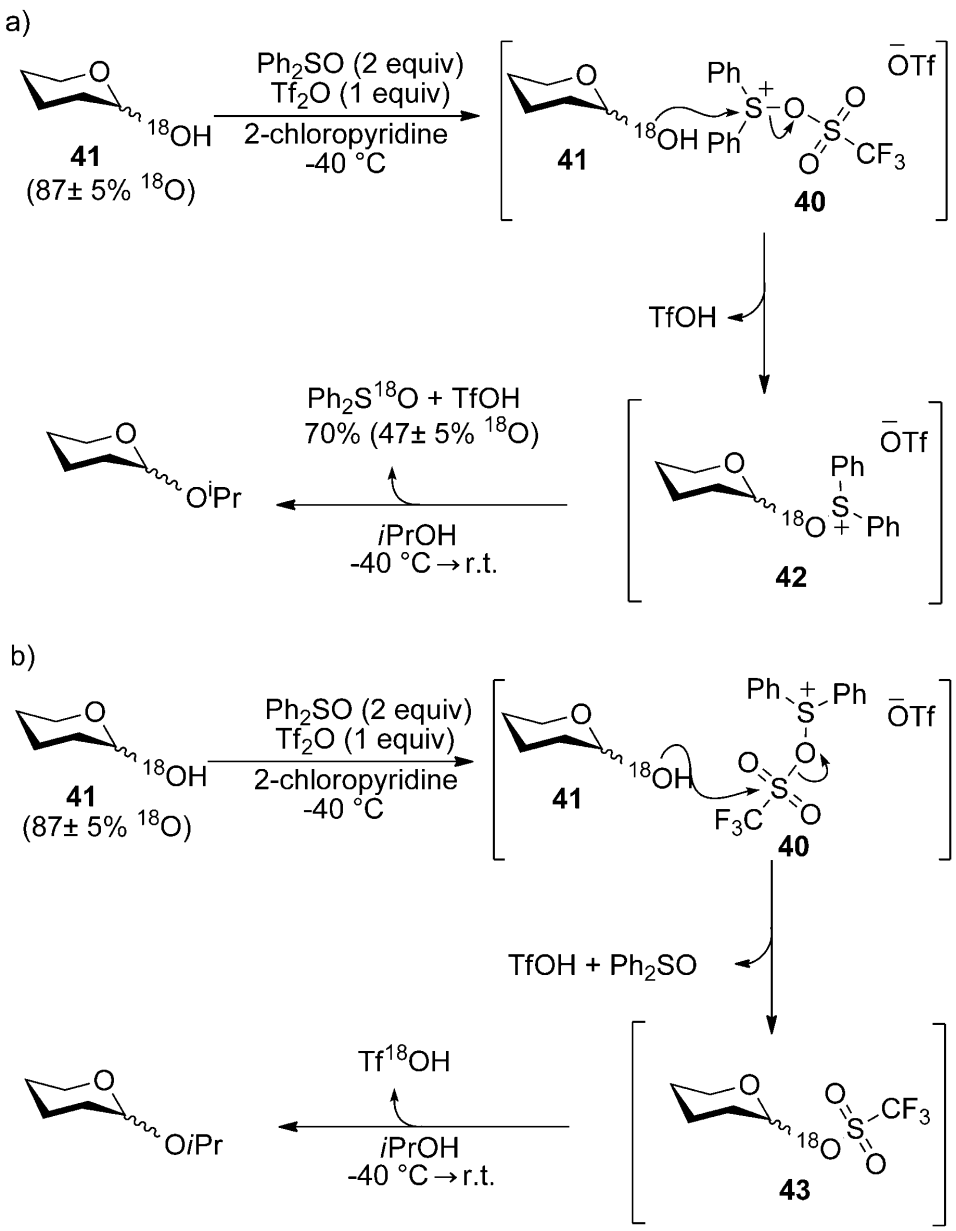
Mechanisms for dehydrative glycosylation involving a) an oxosulfonium species **42** or b) a glycosyl triflate **43**.

The first step of the mechanism is assumed to be activation of Ph_2_SO by triflic anhydride to give trifloxysulfonium ion **40**. This species could then react with hemiacetal **41** through its S^IV^ centre to afford an oxosulfonium intermediate **42** (Scheme 14 a), or through its S^VI^ centre to afford glycosyl triflate **43** (Scheme 14 b). The near quantitative incorporation of the label into recovered Ph_2_SO (47±5 ^18^O‐incorporation, as 2 equiv of Ph_2_SO were used) ruled out the pathway involving glycosyl triflate **43** (Scheme 14 b). ^1^H NMR spectroscopy was used to identify the presence of an oxosulfonium triflate species and a glycosyl pyridinium species as reaction intermediates. The analogous glycosyl triflate previously synthesised by Crich and co‐workers^19^ was not observed in the reaction mixture. The authors noted the observed formation of glycosyl pyridinium species does not necessarily imply it is a reactive intermediate involved in glycoside formation.

Following the initial studies by Gin and co‐workers^29^, ^30^ into the use of sulfoxides in dehydrative glycosylations, the method was utilised in various other examples^31^ including in the efficient synthesis of sialosides.^32^

### Sulfoxide covalent catalysis

**Scheme 15:**
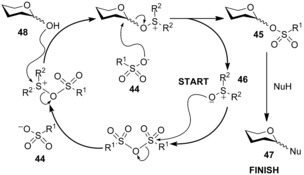
Catalytic cycle for sulfoxide covalent catalysis.

Mechanistic studies into the dehydrative glycosylation (vide supra) suggested the possibility of using catalytic amounts of Ph_2_SO in the reaction; however, attempts to reduce the amount of Ph_2_SO were plagued by self‐condensation of the sugar.30a To circumvent this problem Gin and co‐workers developed a catalytic protocol using a nucleophilic sulfonate counteranion **44** that reacted to form an anomeric sulfonate **45** as a “resting state” for the activated hemiacetal (catalytic cycle, Scheme 15).^33^

**Scheme 16:**
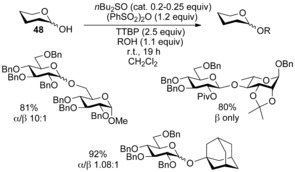
Sulfoxide covalent catalysis with dibutyl sulfoxide and diphenyl sulfonic anhydride

For the protocol to work catalytically the sulfonate counteranion needed to be nucleophilic enough to displace/regenerate the sulfoxide **46**, while the anomeric sulfonate **45** had to be reactive enough to afford glycosides **47**, but also stable enough to prevent self‐condensation with the hemiacetal **48**. Screening identified dibutyl sulfoxide and diphenyl sulfonic anhydride as the ideal combination for glycosyl sulfoxide‐based covalent catalysis (Scheme 16).^33^

An elegant and exhaustive labelling study^34^ was undertaken to confirm the postulated mechanism, using dynamic ^18^O‐label monitoring by low‐temperature ^13^C NMR spectroscopy.^35^

## Sulfoxide‐based activation of glycal donors

**Scheme 17:**
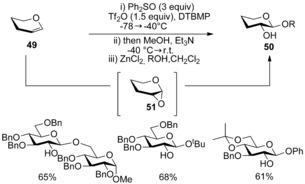
Activation of glycal **50** using Ph_2_SO and triflic anhydride.

Glycal donors **49** had previously been activated in a two‐step procedure using oxidising agent dimethyldioxirane (DMDO)^36^ to afford C(2)‐hydroxy pyranosides **50**. Gin and co‐workers extended their use of sulfoxides as activating agents to achieve the same goal in a one‐pot process.^37^ The combination of Ph_2_SO and triflic anhydride (2:1 ratio) facilitated the formation of 2‐hydroxy pyranosides **50** from glycal donors **49**, by a complex oxidative mechanism that was thought to proceed via an 1,2‐anhydropyranose intermediate **51** (Scheme 17).

**Scheme 18:**
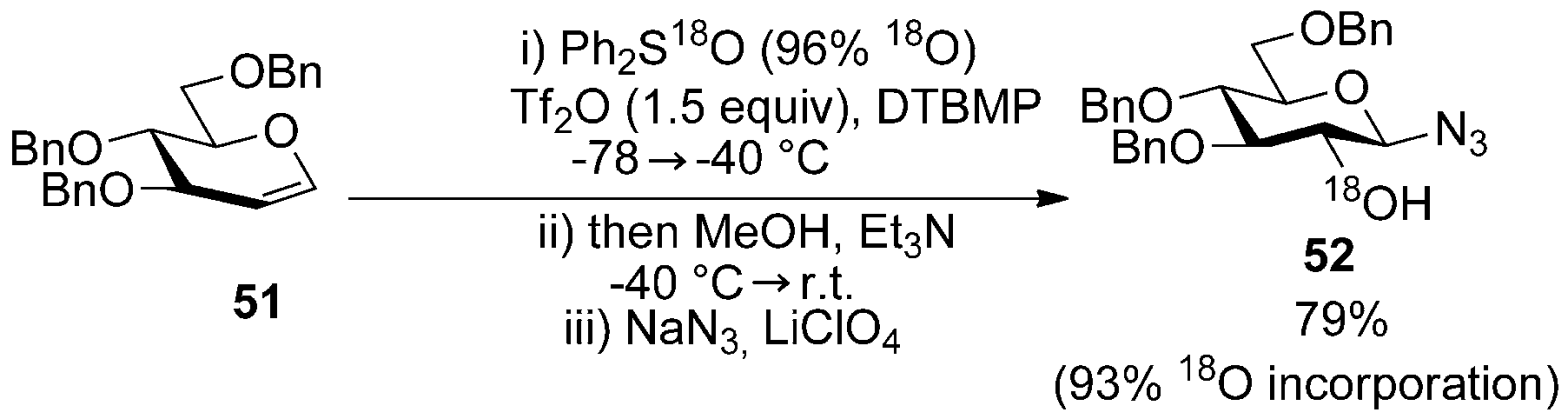
Labelling study using ^18^O‐labelled Ph_2_SO (96 % ^18^O‐incorporation).

The mechanism of the glycosylation reaction was again elegantly dissected using labelling studies.^38^ Transfer of the ^18^O label from Ph_2_SO to C(2)‐OH was observed (Scheme 18).

**Scheme 19:**
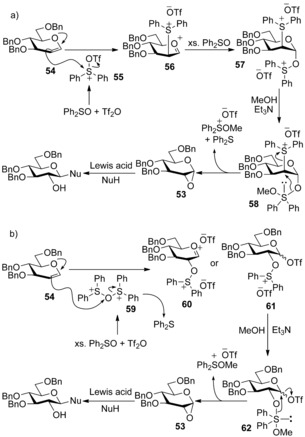
a) Proposed mechanism for glycal activation, incorporating disulfonium species **57**. b) Proposed mechanism for glycal activation, incorporating C‐2‐oxosulfonium dication **60**.

In addition to ^18^O‐transfer from the sulfoxide, the authors observed formation of diphenyl sulfide (0.7 equivalents) and the formation of 1,2‐anhydropyranose **53** as an intermediate following methanol addition (by ^1^H NMR). Therefore, two possible mechanistic pathways were proposed (Scheme 19 a,b).

In mechanism a (Scheme 19 a) the glucal donor **54** is activated by diphenylsulfonium ditriflate **55**, before excess Ph_2_SO reacts with sulfonium species **56** to afford disulfonium species **57**. On addition of methanol, the σ‐sulfurane intermediate **58** 39 forms and subsequently fragments with expulsion of diphenyl sulfide to afford 1,2‐anhydropyranoside **53**. The approach of diphenylsulfonium ditriflate **55** to the β‐face of the glycal is ultimately responsible for the stereocontrol in the glycosylation reaction. Alternatively, in mechanism b (Scheme 19 b), the excess Ph_2_SO gives rise to an oxygen‐bridged disulfonium salt **59**. Attack by the glucal donor **54** at the bridging oxygen atom would afford C‐2‐oxosulfonium dication **60** (or the analogous pyranosyl triflate **61**). On addition of methanol, σ‐sulfurane intermediate **62** forms and affords 1,2‐anhydropyranose **53** by fragmentation. The stereocontrol of the reaction is now governed by approach to the least sterically hindered α‐face by oxygen‐bridged disulfonium salt **59**.

**Scheme 20:**
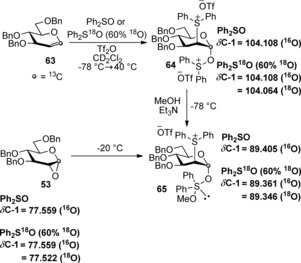
^13^C NMR tracking of the ^18^O‐label position relative to the ^13^C‐label in the activation of glucal **63**.

The key difference between mechanisms a and b is that the oxosulfonium species is either connected to C‐1 (Scheme 19 a) or C‐2 (b). This difference in connectivity was exploited in order to determine which mechanistic pathway was traversed.^38^ When using ^13^C‐1‐labelled glucal donor **63** in a ^13^C NMR tracking experiment, small perturbations in signals were measured when the ^13^C label was directly connected to an ^18^O‐label (Scheme 20).^35^ A comparison of the C‐1 signals using unlabelled Ph_2_SO and labelled Ph_2_SO (60 % ^18^O‐incorporation) made it possible to distinguish whether the disulfonium species **64** and C‐1 σ‐sulfurane intermediate **65** postulated in mechanism a (Scheme 19 a) truly existed. Using labelled Ph_2_SO (60 % ^18^O‐incorporation) perturbation in the C‐1 signal of the first observed glycosyl intermediate established connectivity between ^13^C and ^18^O, consistent with glycosyl oxosulfonium species **64**. After the addition of methanol, perturbation in the C‐1 signal was also observed, consistent with putative C‐1 σ‐sulfurane intermediate **65** which then fragmented to form 1,2‐anhydropyranoside **53** at −20 °C (Scheme 20); a small variance in *δ*C‐1 (^16^O) shift for **65** was noted when using unlabelled or partially labelled ^18^O‐diphenyl sulfoxide, however two signals, for both the ^16^O and ^18^O‐isotopes, are unequivocally observed in the latter case).

The data from this labelling experiment, therefore, inferred that the reaction proceeded by mechanism a (Scheme 19 a). Identical experiments using the analogous ^13^C‐2‐labelled glucal also confirmed a lack of connectivity between ^13^C‐2 and ^18^O, therefore discounting mechanism b (Scheme 19 b) as a possibility.

## Sulfoxide‐based activation of thioglycosides

**Scheme 21:**
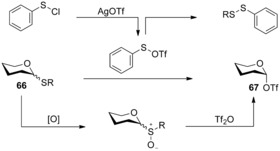
Synthetic routes to a glycosyl triflate **67** species.

The combination of sulfoxide reagents and triflic anhydride has also been applied to the activation of thioglycoside donors. In the pursuit of an expedient route to the aforementioned reactive glycosyl triflate intermediate **17** (Scheme 6), Crich and co‐workers identified electrophilic benzene sulfenyl triflate (PhSOTf) as an effective reagent for the activation of armed and disarmed thioglycosides.^21^ In situ generation of PhSOTf (from benzene sulfenyl chloride (PhSCl) and silver triflate) and subsequent thioglycoside **66** activation provided access to glycosyl triflates **67** quantitatively at low temperatures. The advantage of this method over the glycosyl sulfoxide approach to glycosyl triflates **67** is the exclusion of the sulfide oxidation step prior to the final glycosylation reaction (Scheme 21).

**Scheme 22:**
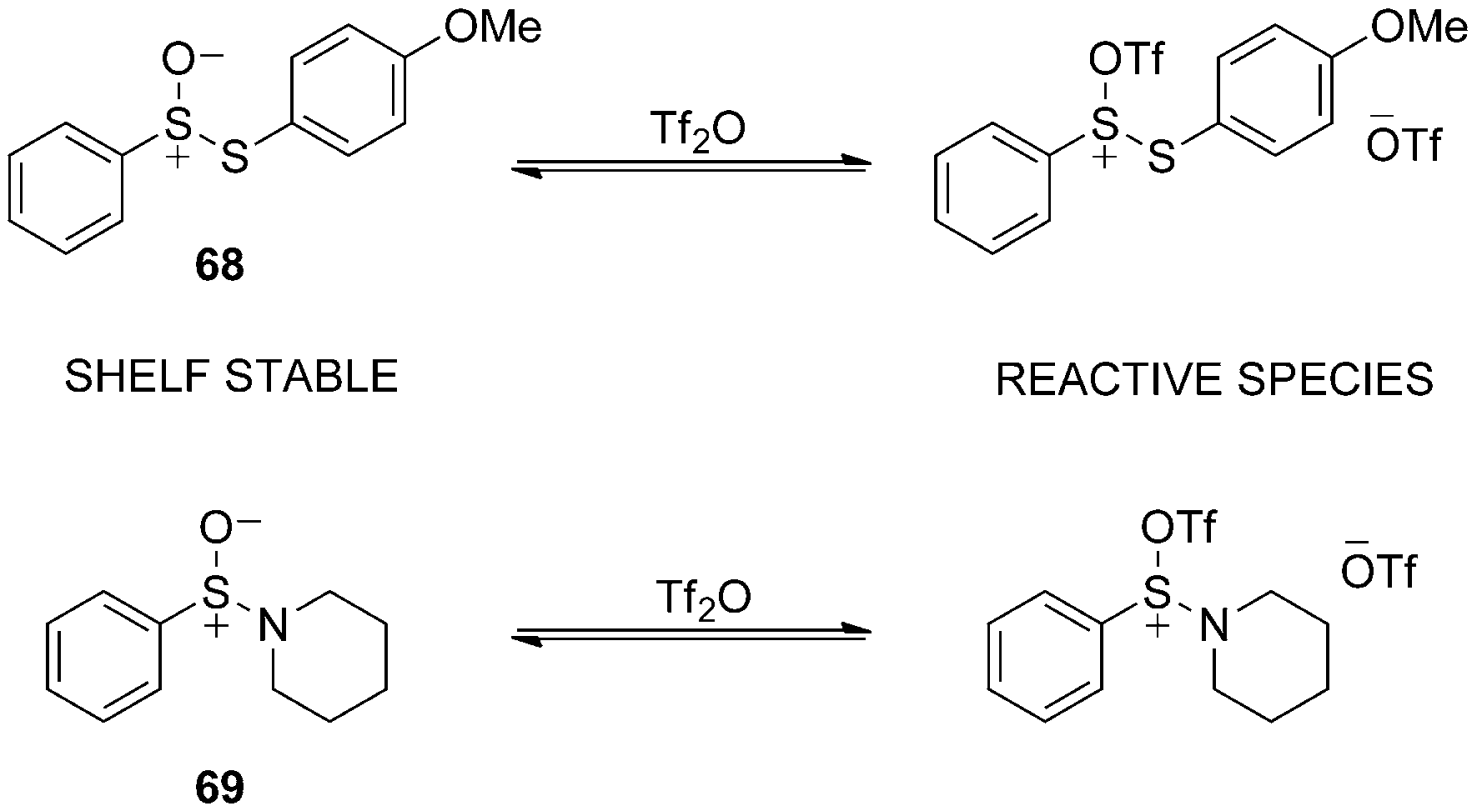
Triflic anhydride activation of MPBT **68** and BSP **69**.

The necessary in situ synthesis of PhSOTf, a result of its marked reactivity and inherent instability, made the process arduous however. To navigate this problem shelf ‐stable *S*‐(4‐methoxyphenyl) benzenethiosulfinate (MPBT) **68** (Scheme 22) was developed and showed reactivity in the activation of armed thioglycosides,^40^ but lacked potency in combination with disarmed donors. An alternative shelf‐stable sulfinamide (BSP) **69** showed much more promise with a range of thioglycoside donors and acceptors, examples included glycosylations with primary, secondary and tertiary alcohols, affording glycosides in excellent yields.^41^

A testament to the efficacy of the BSP/triflic anhydride activation of thioglycosides is the wealth of examples in the literature 24c ,^42^. These notably include use in a one‐pot “reactivity‐based” synthesis of a Fuc‐GM_1_ oligosaccharide,^43^ used with 2,3‐oxazolidinone *N*‐acetyl glucosamine donors^44^ and the activation of 2‐dialkyl phosphate thioglycoside donors.^45^

**Scheme 23:**
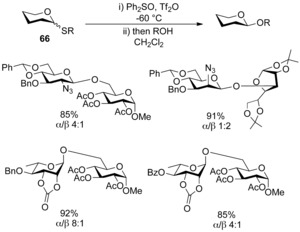
Ph_2_SO/triflic anhydride activation of thioglycosides **66**.

Despite the obvious utility of the activation strategy, attempts to glycosylate unreactive 2,3‐carbonate‐protected rhamnopyranoside donors were unsuccessful using either MPBT or BSP/triflic anhydride. To solve this problem van der Marel and co‐workers intuitively^29^, ^37^ opted to use a combination of Ph_2_SO/triflic anhydride as a promoter, and discovered an even more potent reagent system for the activation of thioglycoside donors.^46^ The replacement of the electron‐donating piperidine ring in BSP with a conventional phenyl group presumably destabilises the adjacent charge on sulfur, and thus increases the reactivity of the sulfonium species. Glycosylation of disarmed donors proceeded in excellent yields (Scheme 23), and selectivities were in line with the proposed formation of glycosyl triflates as intermediate species in the glycosylation reaction.

**Scheme 24:**
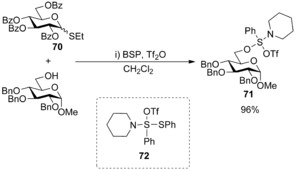
Formation of byproduct **71** and **72**. BSP=1‐benzenesulfinyl piperidine.

Attempts to activate thioglycoside **70** with Ph_2_SO/triflic anhydride or BSP/triflic anhydride in the presence of glycosyl acceptors were unsuccessful as the reactive alcohol sequestered the activating sulfonium species to afford proposed byproduct **71** (Scheme 24),^47^ reiterating the necessity of pre‐activation of the donor. Similarly, chemoselective glycosylations were initially plagued by putative transient species **72**, formed on activation of a thiophenyl donor.46a Yields were low as the disaccharide products formed were activated by sulfonium triflate species **72** and subsequently hydrolysed on workup. Yields could be increased, however, by the addition of triethyl phosphite (TEP) as a reagent to quench the sulfonium triflate species **72** at low temperature before decomposition could take place. A range of other glycosidic transformations have also been effected using thioglycosides in combination with Ph_2_SO/triflic anhydride.^48^ An impressive example illustrated the advantage of Ph_2_SO over the less reactive BSP in conjunction with triflic anhydride. The former was the only reagent successful in the glycosylations of 5‐*N*‐7‐*O*‐oxazinanone‐protected sialoside donors,^49^ and more conventional peracetylated thiosialoside donors were also efficiently activated with Ph_2_SO/triflic anhydride to afford sialosides in excellent yields and α‐selectivities,^50^ with excess Ph_2_SO essential to suppress problematic glycal formation.^51^ In this example, the authors observe formation of oxosulfonium salts at low temperature and propose glycal formation by elimination of the C‐2‐oxosulfonium leaving group is reduced in these intermediates.

## Stereochemical preferences of glycosyl sulfoxides

**Scheme 25:**
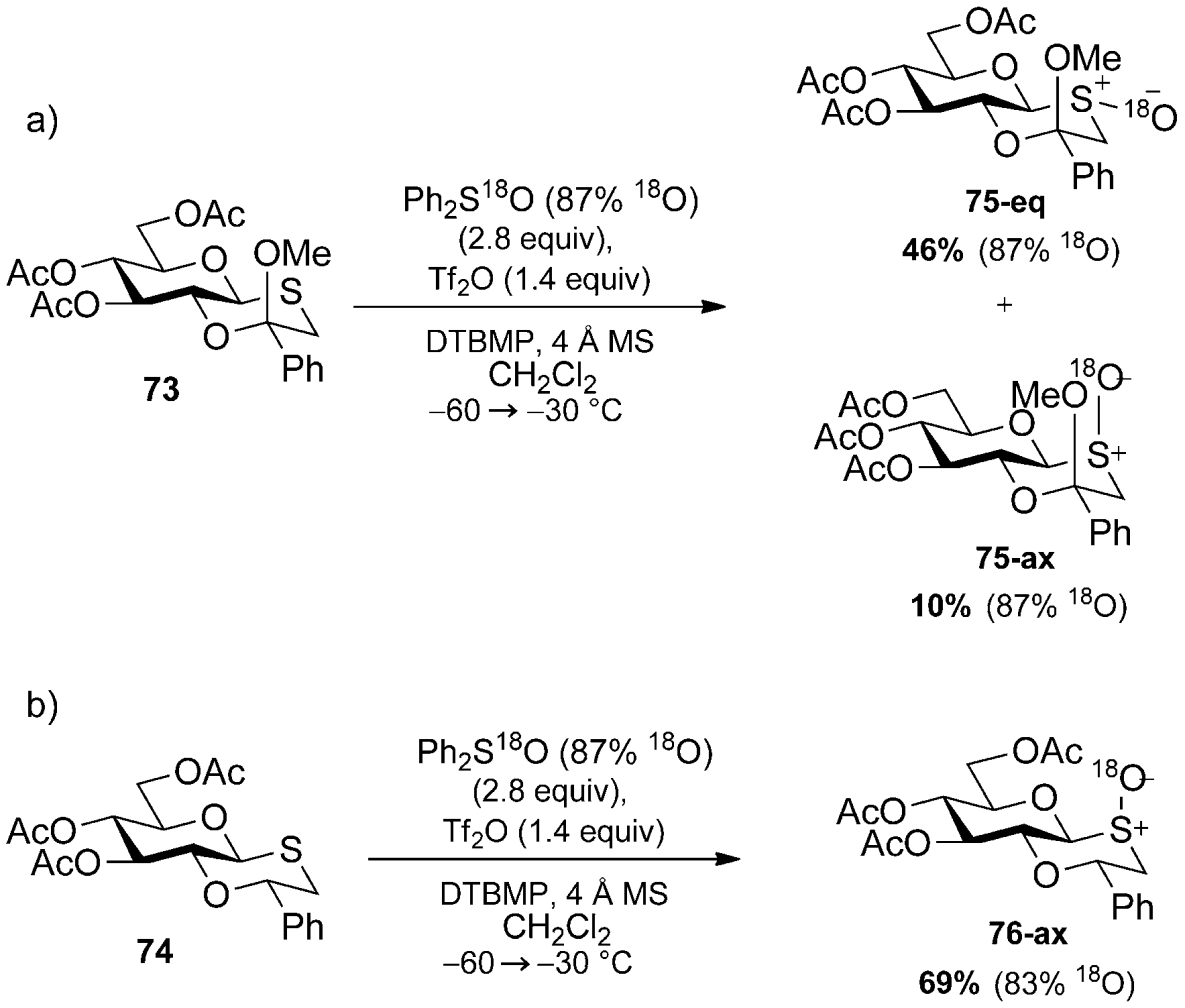
Stereoselective oxidation of glycosyl oxathianes using isotopically labelled Ph_2_S^18^O/Tf_2_O. Reproduced from ref. 47.

Although a lack of detailed studies have been reported on the activation of thioglycosides by sulfonium triflate species, the observations discussed vide supra implied that glycosyl sulfides attack the S^IV^ centre of sulfonium triflate species, or similar reactive intermediates. We provided further strong evidence that this is the case and also gained insight into the stereochemical preferences governing glycosyl sulfoxide formation in a novel transfer sulfoxidation reaction, by once again using the glycosyl oxathiane as a scaffold for serendipitous mechanistic explorations.^52^ When Ph_2_SO/Tf_2_O activation of the ring sulfur in the oxathiane **73**/**74** was attempted, hopeful of stereoselective glycosylation, we were instead surprised to observe stereoselective oxidation to the oxathiane‐*S*‐oxide **75**/**76** (Scheme 25). DFT calculations indicated that the most‐stable stereoisomer was formed preferentially when starting from both oxathiane ketal **73** and oxathiane ether **74**, while low‐temperature ^1^H NMR also demonstrated that the product was formed within minutes at −60 °C in the absence of adventitious water or alcohol. We hypothesised that the reaction must proceed through a novel sulfoxide transfer mechanism after isotopic labelling studies using Ph_2_S^18^O (87 % labelled) unequivocally proved the oxygen in the sulfoxide product originated from Ph_2_SO (Scheme 25).

**Scheme 26:**
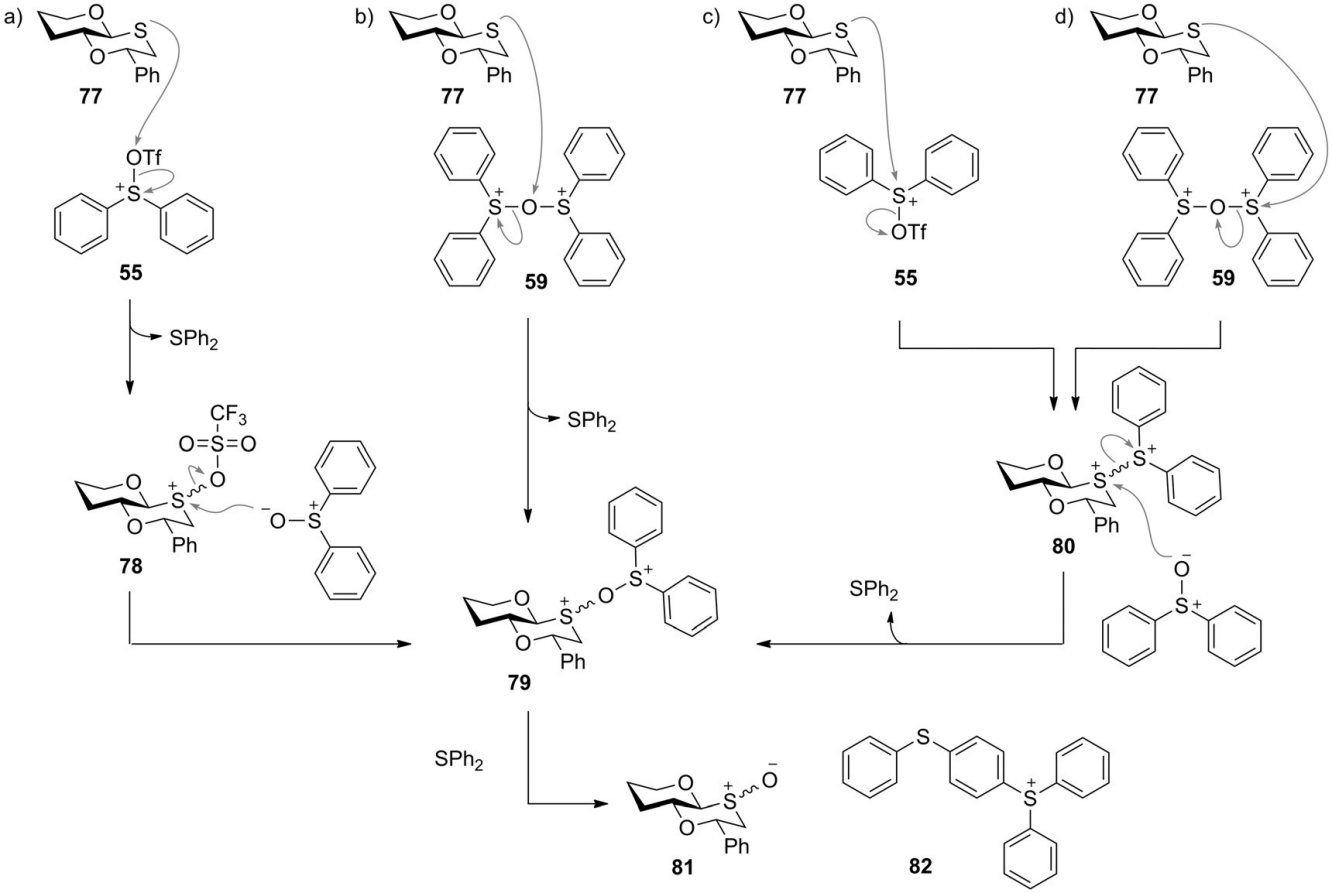
a–d) Possible reaction pathways for the oxidation of generic oxathiane **77**. Mechanisms are depicted as S_N_2 processes for simplicity, although it is likely that some mechanisms may proceed via sulfurane intermediates. Reproduced from ref. 52.

Further detailed ^18^O‐isotopic labelling studies provided evidence for a number of steps that must occur during the sulfoxidation reaction, including that the first committed step in the mechanism must be the reaction of the oxathiane sulfur atom with an activated Ph_2_SO species and a Ph_2_SO oxygen atom must become covalently bound to the oxathiane sulfur atom. Although we were never able to observe or isolate diphenyl sulfide from the sulfoxidation reaction, the quantitative formation of triaryl sulfonium salt **82** (Scheme 26) was confirmed by HPLC mass spectrometric comparison of the crude product mixture with authentic samples of sulfonium salt **82** of known concentration, thus proving diphenyl sulfide must also be produced during the reaction and then react with some activated Ph_2_SO species to produce the triarylsulfonium salt byproduct. Several mechanistic pathways could be proposed and were consistent with these observations (Scheme 26).^52^ In the first (Scheme 26, a), oxathiane **77** initially attacks an electrophilic oxygen atom in triflyloxy sulfonium ion **55** to produce activated oxathiane **78** and diphenyl sulfide. Activated oxathiane **78** could then react with the excess Ph_2_SO to provide oxodisulfonium ion **79**. Similarly, **79** could also be formed by an alternative pathway (b) which also involves reaction at an electrophilic oxygen atom, but on this occasion dication **59**. However, based on literature precedent, vide supra, we deemed routes (a) and (b) to be less likely than attack at the softer electrophilic sulfur atoms in intermediates **55** and **59** (Scheme 26 c,d). If oxathiane **77** were to react at the sulfonium centres of cation **55** (route c) or dication **59** (route d), a dithiadication intermediate **80** would be produced (although seemingly unlikely, intermediate dithiadications have been synthesised previously by reaction between a sulfide and an activated sulfoxide).^33^ Subsequent Ph_2_SO attack at the oxathiane sulfur atom of the dithiadication would then afford oxodisulfonium ion **79**. Thus, regardless of the early steps in the reaction, all pathways converge on oxodisulfonium ion **79**. The final step in the reaction is then a quench of the oxodisulfonium ion by diphenyl sulfide to afford the oxathiane‐*S*‐oxide **81** and triaryl sulfonium ion **82**. We favoured route (d) as the pathway for the formation of the dithiadication, which involves attack on the dication **59**—first, postulated by Gin and co‐workers (Scheme 19) as the reactive intermediate in a 2:1 Ph_2_SO/Tf_2_O activation mix, and then confirmed by our own experiments in this study using ^19^F NMR and ^18^O‐labelling studies. Extension of the labelling studies to a simple non‐glycosyl oxathiane, demonstrated that the stereoselective sulfoxidation was not limited to substrates containing a sugar ring that have the ability to interconvert between axial and equatorial‐orientated intermediates through anomeric bond breaking and generation of an oxacarbenium ion, followed by bond rotation and then intramolecular ring closing. It must therefore also be possible for the axial and equatorial activated sulfoxide intermediates to also interconvert through an intermolecular attack of Ph_2_SO on the activated oxodisulfonium ion **79**, where the lowest‐energy stereoisomer is quenched to afford the lowest‐energy sulfoxide (Scheme 26).

**Scheme 27:**
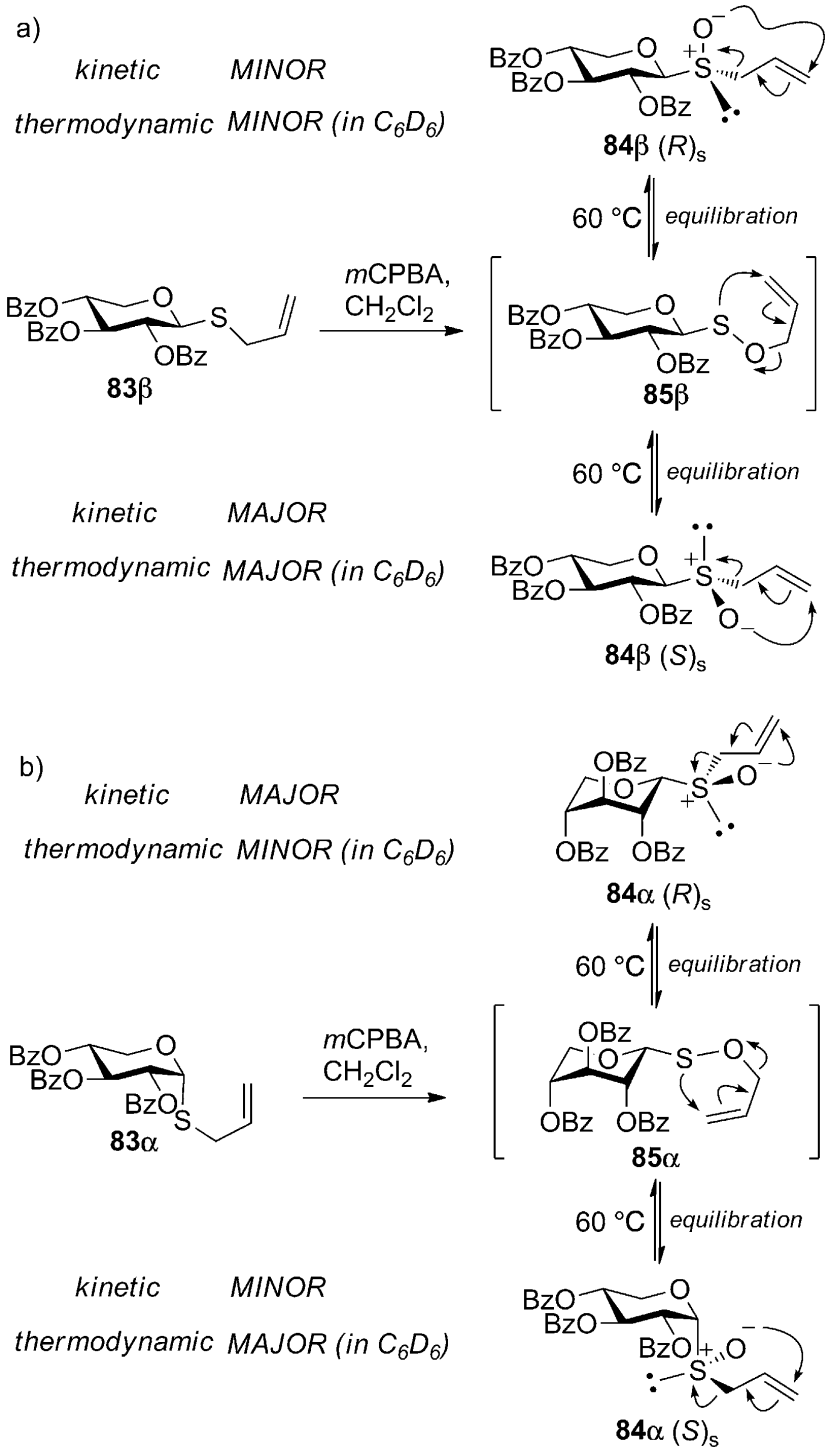
An allyl sulfoxide–sulfenate rearrangement is utilised to probe the kinetic and thermodynamic preferences of sulfoxide formation and equilibration from a) β‐thioxyloside **83 β** and b) α‐thioxyloside **83 α**. *m*CPBA= *meta*‐chloroperoxybenzoic acid.

A number of other detailed mechanistic studies have also been used to dissect some of the more nuanced stereochemical preferences observed in glycosyl sulfoxide formation.^53^ Including Crich and co‐workers^54^ who established inherent stereochemical trends in the oxidation of thioglycosides. The authors concluded that (*R*)_s_ sulfoxides are strongly favoured when axial‐(α)‐thioglycosides are oxidised, as the *exo*‐anomeric effect leads to shielding of the of pro‐*S* sulfur lone pair under the ring and exposes the pro‐*R* lone pair to the solvent, whereas equatorial‐(β)‐thioglycosides afford sulfoxide diastereomers with reduced inherent substrate stereocontrol, only weakly favouring the (*S*)_s_ sulfoxide. An example of the dominance of this stereochemical preference observed for axial‐(α)‐thioglycoside oxidation was noted in the preferential formation of an α‐xylopyranosyl sulfoxide in a seemingly unlikely inverted ^1^ *C*_4_ chair conformation. To investigate this preference Crich deployed a glycosyl allyl sulfoxide‐sulfenate rearrangement to probe the kinetic and thermodynamic preferences of sulfoxide formation from thioxylosides. As expected oxidation of β‐thioxyloside **83 β** preferentially afforded the (*S*)_s_ sulfoxide **84 β** (*S*)_s_ as the major (kinetic) product (Scheme 27 a), while the α‐thioxyloside **83 α** afforded the inverted ^1^ *C*_4_ conformer of (*R*)_s_ sulfoxide **84 α** (*R*)_s_ as the major (kinetic) product (Scheme 27 b). In the former β**‐**series, following thermal allyl sulfoxide **84**‐sulfenate **85** rearrangement in deuteriobenzene, the thermodynamic product proved to be the same as the kinetic product. However, following thermal equilibration of the latter ^1^ *C*_4_ conformer of the sulfoxide **84 α** (*R*)_s_, conversely thermodynamic reversion to the minor kinetic product **84 α** (*S*)_s_ occurred.

The observation that the kinetic sulfoxide **84** **α** (*R*)_s_ exists in the triaxial inverted ^1^ *C*_4_ conformer is explained by the authors as a preference for minimising repulsions between the sulfoxide (*S*)‐*O* and *C*2‐*O*2 dipoles, which are unfavourably aligned in the minor ^4^ *C*_1_ conformer of the (*R*)_s_ diastereomer, but following thermodynamic equilibration to the **84** **α** (*S*)_s_ diastereomer, the preference to ring flip is obviated by a lack of dipole repulsion, meaning **84** **α** (*S*)_s_ exists in the expected ^4^ *C*_1_ conformer.

α‐Thioglycosides and analogous α‐sulfoxides of *S*‐phenyl mannoazide uronate donors were also shown to exist primarily in the ^1^ *C*_4_ confirmation,^55^ as opposed to the corresponding β‐thioglycoside/sulfoxide anomers that adopt a ^4^ *C*_1_ chair in line with the observations made for xylopyranosyl sulfoxides.

## Conclusions

Since their first deployment as an anomeric leaving group over 25 years ago, sulfoxides have become increasingly attractive to synthetic carbohydrate chemists because of their penchant for facilitating interesting and unexpected transformations. As examples of such transformations in the literature have multiplied, so has the ability of chemists to harness and direct this complex reactivity. This has led to the emergence of significant roles for sulfoxides as mediators in a range of innovative mechanistic strategies for probing glycosylation and other cognate reactions, including the development of cation clocks, mass spectrometry and ^13^C NMR isotopic‐labelling studies, and DFT molecular‐modelling studies. Feedback from these mechanistic studies has in‐turn led to improvements in the reactivity, and anomeric stereoselectivity of sulfoxide glycosyl donors for the synthesis of challenging and complex oligosaccharides, as well as a panel of increasingly potent thioglycoside activators for the synthesis of biologically important deoxy sugars, among others. These pioneering studies have also begun to influence the manner in which carbohydrate chemists approach and rationalise glycosylations using other classes of glycosyl donor.

## Biographical Information

*Martin Fascione received his Ph.D. from the University of Leeds in 2009, working under the tutelage of W. Bruce Turnbull on the stereoselective synthesis of 1,2‐*cis*‐glycosides. Following a postdoctoral period in Leeds, he was then awarded a Marie Curie International Outgoing Fellowship to study the mechanisms of carbohydrate‐processing enzymes with Professor Steve Withers, FRS, at the University of British Columbia in Vancouver, Canada (2012–2013) and Professor Gideon Davies, FRS, FMedSci, at the University of York, UK (2013–2014). In August 2014 he took up a lectureship in the York Structural Biology Laboratory, within the Department of Chemistry. His research interests include chemical glycobiology, synthetic carbohydrate chemistry and the chemical/enzymatic modification of proteins*.

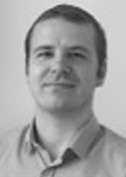

## Biographical Information

*Robin Brabham was born in Southampton (UK) in 1993, and was awarded a MChem degree from the Department of Chemistry at the University of York, UK (July 2015). In October 2015 he commences Ph.D. studies in the Fascione group. Robin's Masters research focussed upon developing new routes to stereoselective glycosyl donors to be deployed in the synthesis of chemical probes with potential use as therapeutic agents*.

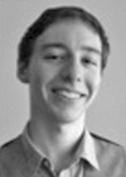

## Biographical Information

*Bruce Turnbull completed his Ph.D. with Prof Rob Field at University of St Andrews in 1998 before taking up a Wellcome Trust International Prize Travelling Research Fellowship at University of California Los Angeles with Prof Sir Fraser Stoddart FRS. He returned to the UK for further postdoctoral studies with Prof Steve Homans at University of Leeds, where he subsequently held a Royal Society University Research Fellowship in the School of Chemistry. He was awarded the Royal Society of Chemistry Carbohydrate Chemistry Award in 2013 for his studies of glycoside synthesis and carbohydrate‐binding proteins. He chairs EU COST Action CM1102 on multivalent glycosystems for nanoscience and has research interests in synthetic glycobiology*.